# Breaking barriers: improving mammography screening accessibility and quality of care for breast cancer women with disabilities in Saudi Arabia

**DOI:** 10.3389/fonc.2024.1398061

**Published:** 2024-11-15

**Authors:** Huda I. Almohammed

**Affiliations:** Department of Radiological Sciences, College of Health and Rehabilitation Sciences, Princess Nourah bint Abdulrahman University, Riyadh, Saudi Arabia

**Keywords:** mammography, screening, accessibility, breast cancer, disabilities, Saudi Arabia

## Abstract

**Introduction:**

Breast cancer screening remains pivotal in early detection and intervention. However, disparities persist, particularly among women with disabilities, necessitating a comprehensive understanding of their screening practices. This study aims to investigate breast cancer screening behaviours in Saudi women with disabilities.

**Methods:**

A cross-sectional study conducted in Saudi Arabia surveyed 307 women with disabilities, evaluating their screening frequency, knowledge of mammography, disability types, and duration. The Statistical Package for the Social Sciences (SPSS) was employed for data analysis.

**Results:**

The study found that 70.4% of participants had irregular breast cancer screenings, and 92.5% lacked tailored information on breast cancer screening. The primary sources of information were support groups (59.3%) and healthcare professionals (25.4%). Significant associations were observed between education levels and awareness of the importance of mammography and the increased risk of breast cancer in individuals with disabilities. Notably, participants with higher education levels demonstrated greater awareness.

**Conclusion:**

The findings highlight substantial gaps in breast cancer screening practices and knowledge among Saudi women with disabilities. There is a critical need for tailored educational programs, accessible information, and targeted awareness campaigns to address these disparities. Enhancing the accessibility of screening services and information for this demographic is essential for improving healthcare equity and outcomes.

## 1 Introduction

Breast cancer screening is essential for early detection and effective management of breast cancer in women, including those with disabilities (1–4).Mammography is one of the most common screening methods used to detect breast cancer in its early stages (5–7). However, it is important to address the unique needs and challenges that females with disabilities may face during the screening process to ensure equitable access to healthcare (8, 9).

It’s crucial to recognise that the needs and abilities of individuals with disabilities can vary widely, so a personalised and patient-centred approach is essential in providing effective breast cancer screening and mammography protection for this population (10, 11).

Barriers to breast cancer screening and mammography protection for females with disabilities include financial, environmental, and physical limitations; psychological barriers; lack of knowledge; fear and embarrassment; anxiety about the examination process; dependency on others; and inadequate understanding of healthcare professionals about their disability (10, 12–15). These barriers contribute to lower participation rates in screening and higher mortality rates for women with disabilities (10, 16). To address these barriers, it is important to remove physical barriers to accessing healthcare services, provide appropriate and less time-consuming examination conditions, and educate healthcare personnel about the needs of individuals with disabilities (17, 18). patient education, reminders, and support from healthcare professionals can encourage participation in screening mammography (19). Strategies to increase the participation of women with disabilities in screening should be developed to eliminate disparities in mammography and Pap testing and reduce the likelihood of breast and cervical cancer diagnoses (20).

Breast cancer screening and mammography protection for females with disabilities can be improved through various best practices (21) One important factor is addressing the barriers that prevent women with disabilities from accessing screening services. These barriers include sociodemographic factors, health insurance limitations, healthcare worker attitudes, and physical barriers (14, 22–24). To overcome these barriers, it is crucial to provide patient education, reminders, and support from healthcare professionals to encourage participation in screening mammography. Additionally, it is important to consider the specific needs of women with disabilities and provide accommodations to make the screening process more accessible and comfortable for them (15, 25). income and education levels have positively influenced screening use, highlighting the importance of addressing socioeconomic disparities in accessing mammography services (26–28). By implementing these best practices, it is possible to improve breast cancer screening rates and mammography protection for females with disabilities (21, 29, 30).

To increase participation in breast cancer screening and mammography protection among females with disabilities, several strategies can be implemented. First, healthcare facilities should ensure they are accessible to individuals with disabilities, remove physical barriers, and provide appropriate examination conditions (31). Second, healthcare professionals should be educated about the specific needs and concerns of women with disabilities, improving their knowledge and understanding (15, 18). Third, efforts should be made to address the psychological barriers faced by women with disabilities, such as fear, embarrassment, and anxiety about the examination process (13, 17, 22).Fourth, educating women with disabilities about the importance of breast cancer screening and the benefits of mammography can help increase their participation (16, 29, 32). Finally, financial and logistical support should be provided to overcome the financial and environmental limitations that may prevent women with disabilities from accessing screening services (10, 33, 34). Cancer screening programs in Saudi Arabia, particularly at the primary care level, have significantly evolved to enhance early detection and treatment outcomes. The Ministry of Health has implemented various initiatives focusing on breast cancer screening through mammography, primarily delivered via primary healthcare centres across the country. Breast cancer is the most prevalent cancer among women in Saudi Arabia, constituting approximately 29% of all female cancer cases. Recent reports indicate that about 19% of eligible women undergo regular mammography screenings, though the screening rate among women with disabilities is significantly lower, highlighting accessibility issues. When evaluating screening strategies, it’s essential to distinguish between population-based and opportunistic screenings. The national breast cancer screening program invites women aged 40-69 for biennial mammography, ensuring equitable access. In contrast, opportunistic screening occurs during routine health visits without structured follow-up, potentially leading to disparities. Statistics show around 5,000 new cases of breast cancer are diagnosed annually, with mammography detecting about 70% of these cases, and the incidence rate is about 24 per 100,000 women. These figures underscore the importance of structured screening programs in improving early detection and outcomes. Addressing barriers faced by women with disabilities is necessary to ensure equal opportunities for early detection.

## 2 Methods

### 2.1 Study design

This cross-sectional study, conducted in Saudi Arabia from May to October 2023, aimed to explore breast cancer screening practices among Saudi women with disabilities. The survey was distributed among Saudi women with disabilities in mammography screening programs.

### 2.2 Sampling, sample size, sampling methods, inclusion and exclusion criteria

A convenience sample of 308 Saudi women with disabilities was included in the study. Sample size calculations, performed using the Open Epi calculator, adhered to a 95% confidence level and considered prevalence rates of breast cancer (ranging from 15% to 50%), necessitating a sample size between 160 and 380 participants. Inclusion was limited to Saudi women with disabilities from various regions of Saudi Arabia who could complete the questionnaire independently or with the assistance of research assistants or relatives. Women who could not complete the questionnaire or refused to participate in the study were excluded. Despite using a convenience sample, efforts were made to ensure that the sample adequately represents the population of Saudi women with disabilities. The sample included participants from various regions of Saudi Arabia, encompassing a broad spectrum of demographic characteristics, such as marital status and education levels.

### 2.3 Survey distribution

The survey was distributed by research assistants proficient in Arabic and English, allowing them to communicate with participants effectively. An online survey platform was also utilised to facilitate accessibility and broader reach among potential respondents. Participants and their relatives received detailed information about the study’s objectives, ensuring informed voluntary participation.

### 2.4 Study instrument

The authors devised the survey using insights from previous studies conducted in English. The authors translated the original content and then reviewed it with two professional translators to ensure accuracy. Additionally, the survey underwent a pilot phase involving three faculty members and three staff from a mammography screening centre to assess readability and comprehension. Insights from this piloting phase were used to refine the survey; however, these details are not included in the results section.

The survey was structured to encompass various segments, systematically gathering essential information from the participants. It commenced with inquiries regarding demographic details, including marital status, education level, and disability type and duration. This section aimed to create a comprehensive profile of the participants, delineating their background and the nature of their disabilities.

Following this, the survey delved into probing the frequency of breast cancer screenings among participants to understand how often they underwent screenings. Subsequently, it delved into assessing their knowledge about mammography and breast cancer, seeking insights into their awareness of the importance of screenings and any specific information tailored for individuals with disabilities. Additionally, it explored the sources through which they obtained information related to breast cancer screening, gauging the prominence of support groups, healthcare professionals, and online resources in disseminating this crucial information.

Utilising a Likert scale and closed-ended queries, the survey comprehensively covered various facets of breast cancer screening, aiming to garner a holistic understanding of the participants’ experiences, awareness, and access to pertinent information.

### 2.5 Hospital selection and participant recruitment

The study was conducted at a prominent hospital in Saudi Arabia known for its comprehensive breast cancer screening program and extensive services for women with disabilities. This hospital was selected due to its high patient volume and established a reputation for providing specialised care to women with disabilities. On average, the hospital receives approximately 200 women for breast cancer screening monthly, ensuring a sufficient pool of potential participants for the study. Women were selected based on their attendance at the hospital’s breast cancer screening program during the study period. Of the 350 women approached, 43 refused to participate, resulting in a response rate of approximately 88%. The high acceptance rate suggests the participants’ strong interest and willingness to contribute to research to improve healthcare outcomes for women with disabilities. By conducting the study at this hospital, the research benefited from a well-established infrastructure and a diverse patient population, further supporting the representativeness and relevance of the study findings.

### 2.6 Ethical approval

Ethical approval from the Institutional Review Board (IRB) at Princess Nourah bint Abdulrahman University in Riyadh City, KSA, was obtained (IRB Log Number: 23-0511).

### 2.7 Data analysis

Data analysis used IBM Corp’s Statistical Package for the Social Sciences (SPSS) version 24. Quantitative variables were presented as percentages, and comparisons were made using the Chi-Square test, supported by associated p-values. Graphical representation was generated using Microsoft Office Excel 2016 in Redmond, Washington. The analysis focused on breast cancer screening practices, mammography knowledge, and information specific to women with disabilities derived from a closed-ended questionnaire response.

## 3 Results

### 3.1 Demographic profile

**Table 1** presents a demographic breakdown of the study cohort. Notably, 21.8% of participants are married, while the majority, 78.2%, are single. Education levels exhibit diversity: 2% lack formal education, 28.7% have an intermediate level, 18.6% hold secondary qualifications, and 50.8% possess a Bachelor of Science degree. The cohort’s diverse marital status and education levels might impact health behaviours and access, influencing study outcomes. These factors could affect decision-making autonomy and health information understanding, demanding careful interpretation of findings, particularly regarding breast cancer screening in disabled women. Analysing these diverse perspectives is crucial for accurate study conclusions.

**Table 1:** Demographic profile: marital status and educational attainment.

		n	%
Marital status	Married	67	21.8
	Unmarried	240	78.2
Level of education	No	6	2
	Intermediate	88	28.7
	Secondary	57	18.6
	B.Sc.	156	50.8

**Table 2** presents the demographic characteristics of disability types and their durations among the study’s participants. The data shows diverse disabilities, primarily in learning (35.2%) and physical (35.8%) categories, followed by sensory (12.4%), intellectual (8.8%), and neuro disabilities (7.8%). Noteworthy subtypes include Down syndrome (4.6%), ADHD (34.2%), and multiple sclerosis (7.8%). Concerning the duration of disability, the majority (64.8%) have experienced their disabilities since birth, with smaller percentages having acquired disabilities within the last ten years (5.0%), between 10-19 years ago (23.3%), or over 20 years (7.1%). These findings emphasise the need for personalised and inclusive approaches in studying mammography screening accessibility for women with disabilities, considering the diverse disability types and durations. Such diversity must be considered when designing interventions to enhance accessibility and care quality for this group.

**Table 2:** Demographics of disability types and durations.

		n	%
Type of disability	Intellectual	27	8.8
	Learning	108	35.2
	Neuro	24	7.8
	Physical	110	35.8
	Sensory	38	12.4
What type of disability do you have	Intellectual -Down	14	4.6
	Intellectual	13	4.2
	Learning-dyslexia	3	1
	Learning -ADHD	105	34.2
	Neuro -MS	24	7.8
	Physical	110	35.8
	Sensory (blindness)	11	3.6
	Sensory (deafness)	27	8.8
How long have you been living with your disability?	Since birth	199	64.8
	Less than ten years	15	5.0
	10-19 years	71	23.3
	More than 20years	22	7.1

**Table 3** reveals the screening frequency of participants. The majority (70.4%) have irregular screenings, only 0.3% undergo annual screenings, and 29.3% are uncertain about their screening frequency. This underscores the need for interventions to promote regular screenings among women with disabilities and highlights potential disparities in healthcare access.

**Table 3:** Breast cancer screening frequency among participants.

		n	%
How frequently do you undergo breast cancer screening?	Annually	1	0.3
	Irregularly	216	70.4
	Not sure	90	29.3

**Table 4** provides an overview of participant knowledge and information regarding breast cancer and mammography. Notably, 41% recognise the importance of mammography, 67.1% have received disability-specific information, and 55.7% are unaware of increased risk. However, only 7.5% have received tailored information about breast cancer screening for individuals with disabilities. This highlights the need for improved education and awareness campaigns for this demographic.

**Table 4:** Knowledge and information on mammography and breast cancer in women with disabilities.

		n	%
Are you aware of the importance of mammography for breast cancer screening in women with disabilities?	No	181	59
	Yes	126	41
Received information specifically tailored to women with disabilities regarding mammography and breast cancer screening.	No	101	32.9
	Yes	206	67.1
Are you aware that individuals with disabilities are at an increased risk of developing breast cancer?	No	171	55.7
	Not Sure	136	44.3
Have you received information about breast cancer screening and protection specific to individuals with disabilities?	No	284	92.5
	Yes	23	7.5

**Table 5** shows information sources for women with disabilities regarding breast cancer screening. Support groups or organisations are the most relied upon (59.3%), followed by healthcare professionals and online sources/websites (25.4% each). Printed materials (2.3%) are the least used source. This highlights the significance of support groups and healthcare professionals in disseminating information to this demographic.

**Table 5:** Information sources for women with disabilities regarding breast cancer screening.

		%	n
Sources of information	Support groups or organisations	59.3	182
	Healthcare professionals	25.4	78
	Online sources/websites	25.4	78
	Printed materials (brochures, pamphlets, etc.)	2.3	7

The chi-square analysis explored the participants’ awareness regarding the importance of mammography in breast cancer screening for women with disabilities and awareness of increased breast cancer risk for individuals based on their level of education and type of disabilities. Chi-square tests show significant associations between education level and awareness of both the importance of mammography X2 (3, N = 307) = 25.957, p = 0.000, and increased breast cancer risk in individuals with disabilities, X2 (3, N = 307) = 172.327, p = 0.000. Notably, it indicates that individuals with no formal education stated that they were aware of the importance of mammography (100%), which the low number of uneducated women could justify. These findings prompt us to interrogate whether participants’ awareness truly encompasses the full spectrum of potential harm and underscore the significance of comprehensive screening. Furthermore, it is imperative to investigate the extent to which awareness intervention programs, typically targeting individuals with limited formal education, have been undertaken in recent years and whether similarly rigorous programs are being implemented for individuals at varying educational levels.

## 4 Discussion

This study highlights many facets of mammography screening accessibility and awareness among women with disabilities. The findings have implications for healthcare interventions, education campaigns, and the development of inclusive and individualised ways to improve access and care quality for this population. By addressing the hurdles and inequities identified in this analysis, efforts can be made to eliminate the difficulties faced by women with disabilities while seeking vital healthcare services (10, 15, 27).

A study on cancer screening for persons with disabilities (PwD) in So Paulo discovered that 44% attended timely screenings, with disability type, gender, and age influencing their decisions (35). Our findings suggest that educational level has a similar effect on women with disabilities’ understanding of the value of mammography in breast cancer screening. Those without formal education had universal awareness (100%), which dropped as education levels increased. These findings are consistent with those of the So Paulo study, underscoring the need for specialised healthcare and educational measures to address the population’s requirements. Addressing disparities and promoting informed healthcare choices are critical (35).

A study by Margaret A. showed that women with disabilities tend to have similar rates of mammogram uptake as women without disabilities, regardless of the severity of functional limitations. However, perceived control positively influences mammogram adherence. This research emphasises the importance of understanding the barriers and enhancing factors affecting breast cancer screening in women with physical disabilities, highlighting the need for further investigation in underrepresented subpopulations (36).

The current study aligned with Joanne E. Wilkinson’s research, which also explored the perceptions of mammography among women with intellectual disabilities. Participants in our study demonstrated motivations such as desiring to fit in and believing in the preventive power of mammography. They expressed feeling adequately prepared and well-informed about the procedure. In contrast, participants in Wilkinson’s study exhibited doubts regarding the preventive aspect of mammography. They faced logistical challenges that left them feeling isolated and uncomfortable during the mammogram, contributing to their reluctance for future screenings. These distinctions underscore the necessity for tailored approaches and comprehensive education to address the unique needs of individuals with intellectual disabilities in the context of mammography screening (37).

The investigation described in this study, as well as the research carried out in Northern Ireland (38), expose discrepancies in breast screening participation among women with disabilities. While the former study concentrates on women with intellectual disabilities, the latter focuses on those with chronic physical disabilities. In both cases, it becomes evident that women with disabilities are less likely to attend breast screening, highlighting the imperative for customised strategies and extensive educational efforts to enhance screening rates within this demographic. This is particularly significant given the expected rise in disability prevalence due to an ageing population (39).

Our study and Alex J. Mitchell’s meta-analysis reveal significant disparities in breast cancer screening among women with mental illness, particularly those with severe mental illness (SMI). These disparities persist even after accounting for emotional distress. This underscores the need for interventions to ensure equitable access to essential healthcare services for individuals with mental health conditions (40).

Both K. Peters’ (13) research and the current study underscore the vital importance of early breast cancer detection and screening for women with specific health conditions. While Peters’ research focuses on environmental, systemic, and process barriers in New South Wales, Australia, the current study addresses disparities in breast cancer screening practices among women with mental illnesses and emotional distress. Both studies emphasise that women with these conditions face significant obstacles in accessing breast cancer screening services. Peters’ research highlights the necessity for personalised care and improved equipment access. In contrast, the current study, alongside Alex J. Mitchell’s meta-analysis, highlights disparities in mammography screening rates among women with mental illness, mood disorders, and severe mental illness (40). These findings collectively stress the importance of tailored interventions and support systems to ensure equitable access to essential healthcare services for these vulnerable populations.

This study has limitations that should be acknowledged. Firstly, using a convenience sample may introduce selection bias and limit the generalisability of the findings, as it may not fully represent the broader population of women with disabilities. Additionally, the sample might not capture the full spectrum of experiences and barriers faced by women with disabilities in different healthcare settings or regions. The reliance on self-reported data could introduce recall bias and inaccuracies, as participants might not accurately remember or report their screening practices and knowledge levels.

Despite these limitations, the study has several strengths. It provides a comprehensive demographic analysis, capturing diverse educational backgrounds, marital statuses, and types of disabilities, which enhances the understanding of the population studied. The study achieved a high response rate of approximately 88%, indicating strong participant engagement and the relevance of the research topic to the target population. By specifically examining breast cancer screening practices among women with disabilities, the study addresses a critical gap in the literature. It highlights the unique barriers and needs of this underserved group. These strengths provide valuable insights into the breast cancer screening practices of women with disabilities in Saudi Arabia while highlighting areas for future research and intervention.

## 5 Conclusion

In conclusion, the present study, along with previous research, highlights the pressing need to address disparities in breast cancer screening practices for vulnerable populations. Our findings, focusing on women with intellectual disabilities, demonstrate that inadequate knowledge, anxiety, and a lack of preparation hinder their engagement with mammography screening. These barriers echo concerns raised in K. Peters’ study (13), which identified environmental and systemic obstacles for women with physical disabilities in Australia. Additionally, our results align with Alex J. Mitchell’s meta-analysis (40), emphasising reduced mammography screening rates among women with mental illness, mood disorders, and severe mental illness.

These findings underscore the critical importance of tailored interventions and comprehensive education to bridge the gap in screening rates and ensure equitable access to essential healthcare services for these populations. Addressing the unique needs and challenges faced by individuals with specific health conditions or disabilities is crucial to improving early breast cancer detection and enhancing overall health outcomes. Future research should continue to explore these disparities and develop targeted strategies to promote effective breast cancer screening practices among these underserved communities.

## 6 Implications

The study highlights the critical need for tailored healthcare education and awareness campaigns for women with intellectual disabilities. Healthcare providers and organisations should develop resources and strategies that account for varying levels of understanding and awareness among this population. Promoting healthcare equity for women with intellectual disabilities requires a multifaceted approach. This involves ensuring that healthcare information is accessible through non-technical language, visual aids, and practical demonstrations. Additionally, involving caregivers in education and awareness initiatives is crucial, recognising their significant role in facilitating access to healthcare. Healthcare providers must enhance their communication skills, emphasising the importance of clear explanations and visual aids to ensure patient comfort and comprehension. Ultimately, addressing disparities in breast cancer screening is essential, and strategies must be implemented to eliminate barriers and enhance access to these critical healthcare services.

## 7 Recommendations

To enhance breast cancer screening among women with intellectual disabilities, a multifaceted approach is recommended. This approach includes the development and implementation of educational programs that emphasise the importance of mammography, the provision of accessible support materials, and the creation of physician training programs to improve communication with this population. Additionally, caregiver workshops can empower support networks, while awareness campaigns can reduce stigma and promote acceptance. Research should continue, exploring various factors that influence screening rates, and policy advocacy is necessary to address the unique needs of individuals with intellectual disabilities within the healthcare system. Collaboration between healthcare providers, disability organisations, and advocacy groups is crucial to establishing a comprehensive support network for these women.

## Data Availability

The raw data supporting the conclusions of this article will be made available by the authors, without undue reservation.
